# Extracellular Vesicles Derived From Mesenchymal Stem Cells (MSC) in Regenerative Medicine: Applications in Skin Wound Healing

**DOI:** 10.3389/fbioe.2020.00146

**Published:** 2020-03-03

**Authors:** Antonio Casado-Díaz, José Manuel Quesada-Gómez, Gabriel Dorado

**Affiliations:** ^1^Unidad de Gestión Clínica de Endocrinología y Nutrición, CIBER de Fragilidad y Envejecimiento Saludable (CIBERFES), Instituto Maimónides de Investigación Biomédica de Córdoba (IMIBIC), Hospital Universitario Reina Sofía, Córdoba, Spain; ^2^Dep. de Bioquímica y Biología Molecular, Campus Rabanales C6-1-E17, Campus de Excelencia Internacional Agroalimentario (ceiA3), Universidad de Córdoba, CIBERFES, Córdoba, Spain

**Keywords:** exosomes, mesenchymal stem cells, skin, wound healing, regenerative medicine, extracellular vesicles

## Abstract

The cells secrete extracellular vesicles (EV) that may have an endosomal origin, or from evaginations of the plasma membrane. The former are usually called exosomes, with sizes ranging from 50 to 100 nm. These EV contain a lipid bilayer associated to membrane proteins. Molecules such as nucleic acids (DNA, mRNA, miRNA, lncRNA, etc.) and proteins may be stored inside. The EV composition depends on the producer cell type and its physiological conditions. Through them, the cells modify their microenvironment and the behavior of neighboring cells. That is accomplished by transferring factors that modulate different metabolic and signaling pathways. Due to their properties, EV can be applied as a diagnostic and therapeutic tool in medicine. The mesenchymal stromal cells (MSC) have immunomodulatory properties and a high regenerative capacity. These features are linked to their paracrine activity and EV secretion. Therefore, research on exosomes produced by MSC has been intensified for use in cell-free regenerative medicine. In this area, the use of EV for the treatment of chronic skin ulcers (CSU) has been proposed. Such sores occur when normal healing does not resolve properly. That is usually due to excessive prolongation of the inflammatory phase. These ulcers are associated with aging and diseases, such as diabetes, so their prevalence is increasing with the one of such latter disease, mainly in developed countries. This has very important socio-economic repercussions. In this review, we show that the application of MSC-derived EV for the treatment of CSU has positive effects, including accelerating healing and decreasing scar formation. This is because the EV have immunosuppressive and immunomodulatory properties. Likewise, they have the ability to activate the angiogenesis, proliferation, migration, and differentiation of the main cell types involved in skin regeneration. They include endothelial cells, fibroblasts, and keratinocytes. Most of the studies carried out so far are preclinical. Therefore, there is a need to advance more in the knowledge about the conditions of production, isolation, and action mechanisms of EV. Interestingly, their potential application in the treatment of CSU opens the door for the design of new highly effective therapeutic strategies.

## Introduction

### Extracellular Vesicles: Definition, Discovery, Classification, Isolation, and Characterization

The word “exosome” is ambiguous, requiring clarification. Thus, it may refer to the “exosome *complex*,” being a proteic (enzymatic) macromolecular machinery, present in archaea and eukaryotic cells, being involved in RNA degradation. On the other hand, the “exosome *vesicle*” is an extracellular particle released from the endosomal compartment of most eukaryotic cells. This review deals with the latter only. The exosome vesicles are a type of extracellular vesicles (EV), which are defined as lipid-bilayer spheroid structures, without replicating capacity, that are released from cells, including both prokaryotes and eukaryotes. That includes from simple unicellular organisms to complex multicellular ones. These structures are involved in intercellular-communication mechanisms, being preserved by evolution. Their functionality has been observed not only between cells of the same organism, but also among cells from different organisms of the same or different species, even involving prokaryotes and eukaryotes (Kim et al., 2015; Gho and Lee, 2017). For instance, inter-kingdom communications have been found between the microbiota and the epithelial cells of the large intestine, contributing to maintain the intestinal homeostasis (Cañas et al., 2018).

The EV were first reported after observing procoagulant platelet-derived particles in normal blood plasma (Chargaff and West, 1946). Such particles were defined as “platelet dust” (Wolf, 1967). The release of vesicles generated after the formation of multi-vesicular bodies (MVB) in reticulocytes was independently reported by two research teams, at the beginning of the 1980s (Pan and Johnstone, 1983; Harding et al., 1984). Later on, the term exosome was coined for these endosomal vesicles (Johnstone et al., 1987). Since then, EV have been purified from different cellular types of mammals. In addition, they have been also found in other biological fluids, like urine, breast milk, blood serum (blood plasma without clotting factors), saliva, and semen (Yáñez-Mó et al., 2015). Interestingly, EV may contain ribonucleic acids (RNA), and in particular microRNA (miRNA) (Ratajczak et al., 2006; Valadi et al., 2007). That sparked the interest on such particles, as mediators or intercellular communications. Thus, research on the EV biology has exponentially increased in the last two decades, including their physiological and pathologic roles *in vivo*. Thus, the PubMed database^1^ showed only four exosome vesicle hits in 1999, increasing to more than 11,000 in February 2020.

**FIGURE 1:**
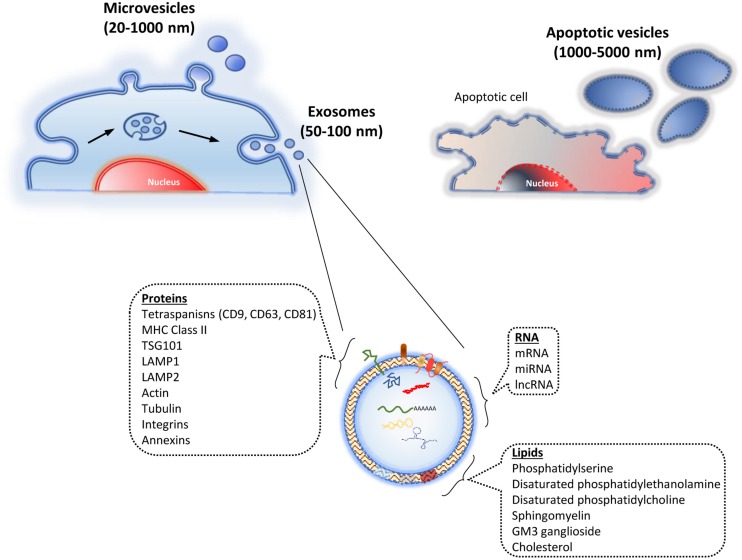
Types of extracellular vesicles. They are classified taking into account their origin. Thus, they include the ones derived from endosomes (exosomes), evagination of the plasma membrane (microvesicles), and vesicles from apoptosis (apoptotic vesicles). The lower part shows the exosome structure, including its main cargo molecules.

The EV have been traditionally classified into four types, mainly taking into account their origins and sizes: (i) endosomal exosomes (50–100 nm); (ii) microvesicles (MV) from the plasma membrane (20–1000 nm); (iii) membrane particles, also from the plasma membrane (50–600 nm); and (iv) apoptotic vesicles from the plasma membrane and endoplasmic reticulum, through apoptotic processes (1000–5000 nm) (Figure 1) (van der Pol et al., 2012). Other authors have classified them into just two types: exosomes, as previously described, and ectosomes (microparticles-MV). The latter ones are derived from the plasma membrane, ranging from 100 to 350 nm (Cocucci and Meldolesi, 2015). Due to the current lack of consensus about the classification and biochemical markers characterizing the different EV types, the International Society for Extracellular Vesicles stated the following in the “Minimal Information for Studies of Extracellular Vesicles 2018” (MISEV2018), in relation to the EV nomenclature: “EV is the preferred generic term for the subject of our investigations, and subtypes should be defined by physical and biochemical characteristics and/or conditions/sources. When other terms are used, careful definition is required” (Théry et al., 2018).

The traditional approach to isolate EV is differential ultracentrifugation. However, new methods have been developed in the last years, like density-gradient ultracentrifugation, allowing to isolate more specific EV populations. Likewise, other technologies have been applied to reduce the isolation time, without expensive specialized equipment. Such later methodologies include the following, among others: (i) exosome precipitation using polymers, like the ExoQuick family of reagents from System Biosciences^2^ (Palo Alto, CA, United States); (ii) immunological methods, to capture and quantify exosomes from different fluids; (iii) size-exclusion chromatography; and (iv) magnetic-activated cell sorting (MACS) (Gurunathan et al., 2019).

Characterization of EV is fundamental to determine their biochemical properties and biological functions. That can be accomplished using different methodologies, allowing to determine size, shape, concentration, contents, and surface biochemical-markers. They include: (i) western blotting; (ii) identification and quantification of nucleic-acid contents, using polymerase chain-reaction (PCR), microarray and second-generation sequencing (SGS) and third-generation sequencing (TGS), sometimes known with the ambiguous name of “next”-generation sequencing (NGS); (iii) lipidomic approaches; (iv) nanoparticle-tracking analyses (NTA) or (v) tunable-resistive pulse sensing (TRPS), both for determination of the size and concentration of particles; (vi) dynamic-light scattering (DLS) or (vii) photon-correlation spectroscopy (PCS), both to measure exosome sizes; (viii) atomic-force microscopy (AFM) or (ix) transmission electron microscopy (TEM), both for visualization and characterization of their structure, morphology, and size; (x) flow cytometry, for the characterization of surface biochemical markers; and (xi) fixation for *in situ* imaging (Choi et al., 2013; Kreimer et al., 2015; Gupta et al., 2019; Gurunathan et al., 2019).

### Biogenesis

The EV cargos depend on the vesicle types, as well as the cells from which they are derived, and their physiological conditions. The main components of the EV are proteins, lipids, and nucleic acids (Figure 1). EV may contain specific groups of cellular proteins, independently of the producing cell. Nevertheless others are secreting-cell-specific peptides. The proteins found in the EV include the ones from the endosome itself, plasma membrane, and cytosol. The proteins from the nucleus, mitochondria, endoplasmic reticulum, and Golgi complex are usually absent in the EV. Interestingly, that shows a specific differential selection of proteins when generating such vesicles (Colombo et al., 2014).

On the other hand, the lipid composition of the EV depends on the cellular types from which they are derived. Their lipid bilayer mainly contains the components from the plasma membrane, but they may be enriched in some of them, including phosphatidylserine, disaturated phosphatidylethanolamine, disaturated phosphatidylcholine, sphingomyelin, GM3 ganglioside, and cholesterol (Choi et al., 2013).

Since the discovery that EV carry nucleic acids (Ratajczak et al., 2006; Valadi et al., 2007), numerous studies have described the presence of different RNA types in such particles. They include messenger RNA (mRNA), miRNA, and non-coding RNA (ncRNA). Again, as with proteins and lipids described above, the comparative analyses of nucleic acids between the cells and the EV generated from them may show differential contents.

**FIGURE 2:**
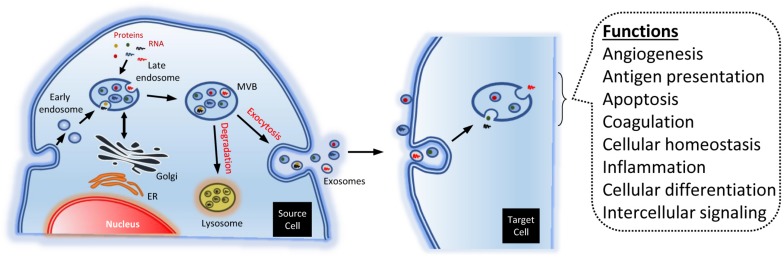
Endosomal biogenesis of exosomes. The endosomes generate multi-vesicular bodies. The latter carry different types of molecules, like RNA and proteins. Such cargos are partially added in a specific way. The MVB may be degraded by the lysosomes, or merge with the plasma membrane, dumping contents to the extracellular space. The exosomes may bind and activate different membrane receptors in the target cells. Alternatively, they can be engulfed, releasing their cargos into the cell. The exosomes may modulate numerous physiological process, though such mechanisms.

The biogenesis of exosomes is due to exocytosis of multivesicular endosomes. Such MVB fuse with the plasma membrane, being released to the extracellular environment. Thus, the exosome biogenesis can be divided into three stages: (i) formation of endocytic vesicles, by invagination of the plasma membrane; (ii) formation of MVB, by inward budding of the endosomal membranes; and (iii) fusion of MVB with the plasma membrane and release of the exosomes (Figure 2) (Colombo et al., 2014).

In many instances, the contents of the MVB are degraded by hydrolases, if the former merge with lysosomes. But, in other instances, some MVB may fuse with the plasma membrane. That allows to release their contents to the extracellular environment (Figure 2). Specific MVB features include the presence of tetraspanins {membrane proteins associated to lysosomes, like lysosomal-associated membrane protein 1, 2, and 3 [LAMP-1, LAMP-2, and LAMP-3, respectively; also known as cluster of differentiation 107a, 107b, and 63 or 208 (CD-107a, CD-107b, and CD-63 or CD-208 antigen), respectively]}, besides other molecules generally present in the late endosomes [e.g., major histocompatibility complex (MHC) class II, in antigen-presenting cells] (Raposo et al., 1996; Colombo et al., 2014).

The best-known mechanism of MVB and exosome generation is the one carried out by the endosomal sorting complex required for transport (ESCRT). It is composed of approximately 30 proteins, generating four complexes (ESCRT-0, -I, -II, and -III) with associated proteins, such as vacuolar protein sorting-associated protein 4 (VPS4), vesicle trafficking 1 (VTA-1), and apoptosis-linked gene 2 (ALG-2)-interacting protein X (Alix), also called programmed cell-death 6 interacting protein (PDCD-6-IP) (Hanson and Cashikar, 2012). The ESCRT-0 complex recognizes and sequesters ubiquitinated proteins in the endosomal membrane. On the other hand, ESCRT-I and -II complexes are responsible for membrane deformation into buds, with sequestered cargos. Finally, ESCRT-III drives vesicle scission (Juan and Fürthauer, 2018). Besides such ESCRT-dependent pathways, other ESCRT-independent mechanisms for EV biogenesis have been described. They involve hydrolysis of sphingomyelin into ceramide, or proteins like tetraspanins, as CD-63 (Andreu and Yáñez-Mó, 2014; Colombo et al., 2014). Tetraspanins are also involved in cargo secretion of EV, as well as the process of uptake by receptor cells (Andreu and Yáñez-Mó, 2014).

### Functions

Accumulating evidence suggests that the EV have a vital role, not only in the regulation of normal physiological processes, such as stem-cell maintenance, tissue repair, and immune modulation (Mistry et al., 2012; Basu and Ludlow, 2016; Chan et al., 2019), but also in the pathology underlying the occurrence of several diseases (El Andaloussi et al., 2013). The EV regulate physiological events and cellular behavior through several mechanisms. For instance, activating membrane receptors through proteic or lipidic ligands, as well as pouring their contents into receptor cells (Figure 2). This way, they can transfer transcription factors, oncogenes, miRNA, mRNA, and even infectious particles (Yáñez-Mó et al., 2015). Because of that, they have been considered signalosomes: multifunctional signaling complexes for controlling fundamental cellular and biological functions (El Andaloussi et al., 2013).

The biological processes involving EV include angiogenesis, antigen presentation, apoptosis, coagulation, cellular homeostasis, inflammation, cellular differentiation, and intercellular signaling. It has been found that the exosomes from progenitor cells stimulate migration, proliferation, and formation of blood vessels in endothelial cells (Yi et al., 2019). The exosome liberation may inhibit or activate apoptosis, depending on their cargos and the receptor-cell types (Xiao et al., 2016; Zhu et al., 2017). The functionality of EV as antigen-presenting helper T lymphocytes is known since the end of the last century. Thus, it has been reported that B lymphocytes secrete vesicles enriched in the MHC class II, with capacity to activate T lymphocytes (Raposo et al., 1996). The EV can work as pro-inflammatory (e.g., when they are produced by cancer cells, synovial fibroblasts, CD-4 + T cells, macrophages, or dendritic cells), or as anti-inflammatory [e.g., when derived from mesenchymal stem cells or mesenchymal stromal cells (MSC), dendritic cells, or T cells]. As a consequence, the EV may play an important role in the genesis or prevention of immunologic diseases, like bowel disease, sepsis, arthritis, diabetes, atherosclerosis, and neurodegenerative ones (Chan et al., 2019).

**FIGURE 3:**
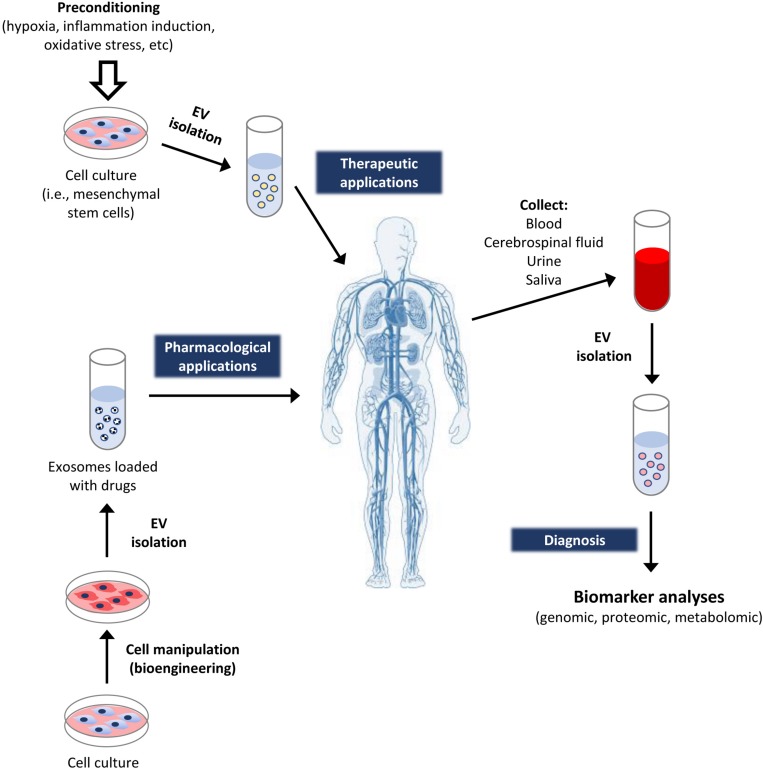
Applications of extracellular vesicles in medicine. The EV obtained from cellular cultures may be applied to patients with specific therapeutic objectives. For instance, the ones derived from cells with regenerative capacity (like MSC) may be used for tissue regeneration. Additionally, the producing cells may be bioengineered to produce EV enriched in some cellular molecules (like specific RNA or proteins), or being loaded with specific drugs. This way, the EV work as vehicles for efficiently targeting the delivery of such molecules to patients. On the other hand, the exosomes can also be isolated from different fluids or tissues of healthy persons, as well as patients suffering different diseases. That allows to identify biomarkers in them, for subsequent diagnostic applications.

The physiological roles of the EV depend on their cargos and their capacity to transfer proteins, nucleic acids, and other molecules into the receptor cells. Fortunately, they can be intravenously given to patients, having the capacity to pass through the blood–brain barrier. Therefore, they have been recently considered as relevant therapeutic tools, with significant potential for the prognosis, diagnosis, and treatment of several pathologies (Gurunathan et al., 2019). This is due to several reasons, including that they can: (i) carry different biomolecules, reflecting the physiological condition of producing cells; (ii) be used as stable biomarkers of pathologies, like cancer (Fu et al., 2018); (iii) move through the body fluids; (iv) be identified from such body fluids, like cerebrospinal, urine, saliva, and blood, facilitating their detection and quantification by non-invasive techniques; (v) be used as therapeutic tools to transport and liberate drugs, including miRNA, short hairpin RNA (shRNA), small-interfering RNA (siRNA), as well as other compounds of pharmacological interest, for the treatment of diseases, like cancer (Di et al., 2018); and last, but not least (vi) be isolated from different cellular types with regenerative and/or anti-inflammatory capacity, with potential of being applied to repair damaged tissues (Figure 3).

Among the most used cellular types for such latter application are the MSC. Indeed, it has been demonstrated that the EV derived from them have an interesting potential for the treatment of cardiovascular diseases, renal, liver, and neural injuries, as well as skin wounds (Yamashita et al., 2018). Due to the interest and multiple studies available about the clinical applications of the exosomes derived from MSC, the main objective of the present work is to show a critical review about their therapeutic properties, including their specific applications for the treatment and healing of skin ulcers.

## Properties and Therapeutic Potential of MSC

The MSC were first described as having a morphology similar to fibroblasts, behaving as colony-forming unit-fibroblast (CFU-F), capable to differentiate into osteoblasts, in guinea-pig bone marrow and spleen (Friedenstein et al., 1970). Subsequently, it was found that such cells can differentiate into different cellular types and tissues of mesenchymal origin, like bone, fat, and cartilage (Pittenger et al., 1999). Interestingly, it has been recently reported that such cells can be also induced to differentiate into cells of endodermic and ectodermic origin, like hepatocytes and neurons, respectively (Lee et al., 2018a; Marei et al., 2018).

The MSC have been isolated from different tissues, including bone marrow, fatty tissue, hair follicle, synovium, umbilical cord, placenta, and periodontal ligament, among others (Huang et al., 2011). Surprisingly, there is not a specific biochemical marker defining the MSC. Therefore, the scientific consensus, as we have previously described, is that they should not exhibit hematopoietic and endothelial markers, like CD-11b, CD-14, CD-31, CD-33, CD-34, CD-45, and CD-133 (Casado-Díaz et al., 2016). Thus, the International Society for Cellular Therapy (ISCT) has defined the minimum MSC features: (i) are plastic-adherent under standard culture conditions; (ii) express CD-73, CD-90, and CD-105, lacking the expression of CD-11b, CD-14, CD-19, CD-34, CD-45, CD-79a, and HLA-DR; and (iii) may differentiate into osteoblasts, adipocytes, or chondrocytes *in vitro* (Dominici et al., 2006). Likewise, it has been proposed that the MSC are involved in maintaining cellular homeostasis in the organism. That is accomplished through tissue regeneration and repair.

The MSC represent a convenient experimental model for studying cellular differentiation events. Likewise, to investigate how some physiological situations, drugs, and other compounds may modulate them. As a practical example, we have used such a model for genome-wide transcriptomic studies during adipogenesis, analyzing how natural products like the oleuropein polyphenol (adipogenesis inhibitor present in the olive oil, among other products) modulates it (Casado-Díaz et al., 2017a, b, 2019). On the other hand, their capacity to differentiate into different cellular types, as well as their anti-inflammatory and immunosuppressive activities (Regulski, 2017), have made them an interesting therapeutic tool in cellular therapy and regenerative medicine, as we have reviewed (Casado-Díaz et al., 2016). Such interesting therapeutic potential is mainly due to the following: (i) can be easily isolated and expanded *in vitro*; (ii) can be cryopreserved once isolated, without significant loss of their therapeutic potential; (iii) show intermediate and low levels of the MHC class I and II molecules, respectively, and therefore, they are hypoimmunogenic; and (iv) can be intravenously administered, efficiently reaching the damaged tissue via the bloodstream.

The interest on the therapeutic potential of the MSC has sparked multiple clinical studies about their applications on different pathologies. They include different types of tumors, multiple sclerosis, amyotrophic lateral sclerosis, stroke, acute and chronic heart failure, diabetes, rheumatoid arthritis and osteoarthritis, osteonecrosis, lumbar intervertebral disc degeneration, Crohn’s disease, kidney and liver chronic disease, sepsis, spinal cord contusions, and critical limb ischemia, among others. Thus, searching for “mesenchymal stem cells” (other terms; no quotes) in the ClinicalTrials.gov database resource of the National Library of Medicine of the United States of America (USA)^3^ showed more than 1000 studies at the time of writing.

## MSC-Derived Extracellular Vesicles for Cell-Free Therapies

Surprisingly, patients inoculated with MSC to promote tissue regeneration showed < 1% of such cells in the damaged tissue after 1 week (Rani et al., 2015; Phinney and Pittenger, 2017). Yet, paradoxically, such strategy has produced positive results in the treatment of several pathologies, favoring tissue regeneration and functionality (Brown et al., 2019). Therefore, it has been suggested that the regenerative effect of the MSC is not mainly due to their capacity to proliferate and differentiate into the required cellular types in the damaged tissue. Instead, their main functionality would stem from their paracrine actions, through the production of different factors (Rani et al., 2015; Marote et al., 2016). Interestingly, such hypothesis is supported by several studies, demonstrating that conditioned media from MSC cultures have a similar regenerative capacity—or even higher—than the MSC themselves. For instance, that has been demonstrated in rodent models of acute myocardial infarction (Gnecchi et al., 2005; El Andaloussi et al., 2013). These results demonstrate the surprising therapeutic relevance of the MSC secretome. In view of these results, Caplan (one of the precursors of the MSC studies) has proposed to rename such stem cells as “medicinal signaling cells” (Caplan, 2010, 2017).

The secretome of the MSC has one free fraction, made of soluble factors and metabolites, as well as other encapsulated into MV, to which the EV belong. Interestingly, it has been found that the latter is the main responsible for the therapeutic properties of the conditioned media from MSC cultures (Kusuma et al., 2017). This way, those EV can regulate different physiological processes, like cellular proliferation, differentiation, and migration (Shimoda et al., 2017; Zou et al., 2018).

The therapeutic features of the MSC EV are mainly due to their immunomodulatory and immunosuppressive activities. This way, they can reduce the levels of cytokines, like interleukin 1 beta (IL-1b) and tumor-necrosis factor alpha (TNF-a), increasing the ones of the transforming growth-factor beta (TGF-b) (Chen et al., 2016). That can be exploited for the treatment of some pathologies, like those in which intense or chronic-inflammation processes may limit or jeopardize healing (Harting et al., 2018). Other relevant and interesting characteristic of the EV from the MSC is their antiaging and antifibrotic activities. Thus, the EV, secreted from both induced pluripotent stem cells (iPSC) and young MSC, reduce the cellular senescence associated to aging MSC cultures, by reducing the intracellular levels of reactive oxygen species (ROS) (Liu et al., 2019). On the other hand, the anti-fibrotic effect of EV can be applied to the treatment of fibrosis in organs such as the liver, heart, lung, and skin (Zhao et al., 2017; Davidson and Yellon, 2018; Mansouri et al., 2019; Rong et al., 2019).

The use of exosomes in therapy has relevant advantages, in relation to complete MSC (Keshtkar et al., 2018). Among them are the following: (i) can be isolated and stored at low temperatures (e.g., –80°C), until needed, without requiring the production of large amounts of cells at the time of inoculation, which is needed for cellular therapy; (ii) their contents are encapsulated and protected from degradation *in vivo* (preventing some of the problems associated with small soluble molecules, such as cytokines, growth factors, transcription factors, and RNA, which are rapidly degraded); (iii) are quite stable, exhibiting a long average life; (iv) can be intravenously injected, reaching distant places, since the vesicles are small and circulate readily, whereas the MSC are too large, and thus may have difficulty circulating through thin capillaries; (v) can pass through the blood-brain barrier; and (vi) have reduced risks of unwanted side-effects, like immune rejection (as said above, they are hypoimmunogenic), cell dedifferentiation, or tumor formation, which can arise after applying exogenous cells (Jeong et al., 2011; Konala et al., 2016; Phinney and Pittenger, 2017; Mardpour et al., 2019).

It is currently estimated that more than 200 preclinic studies are being carried out with different animal models, with promising results (Elahi et al., 2019). The pathologies treated this way include immunologic, cardiovascular, renal, musculoskeletal, and neuronal ones, besides cancer and chronic skin ulcers (CSU), sometimes known as chronic cutaneous ulcers (CCU) (Rani et al., 2015; Marote et al., 2016; Elahi et al., 2019). In relation to the use of MSC-derived EV for skin ulcer healing—which is the main topic of this review—several studies have shown that they may contribute to accelerate skin wound healing and scar reduction. They include the ones derived from different sources, like adipose mesenchymal cells, human amniotic epithelial cells, endothelial progenitor cells (EPC) from human umbilical cord blood, human iPSC-derived MSC (hiPSC-MSC) and cardiosphere-derived cells, among others (Liu et al., 2018).

Clinical trials have recently started using EV from MSC. Thus, searching for “exosome mesenchymal stem cells” (other terms; no quotes) in ClinicalTrials.gov (see above) showed seven studies at the time of writing. One of them uses exosome identification as diagnostic tool. The other are related to their use in acute ischemic stroke, healing of large and refractory macular holes, β-cell mass regeneration in type 1 diabetes mellitus, dystrophic epidermolysis bullosa, and chronic ulcer wounds.

The properties of the EV derived from the MSC depend on the origin and culture conditions of such cells. Thus, differences have been described between the secretome of the human bone-marrow mesenchymal stem cells (BM-MSC), adipose-tissue stem cells (ATSC), and umbilical-cord perivascular cells (UCPC) (Pires et al., 2016). Cells grown under hypoxia produce exosomes with enhanced angiogenic, regenerative, and immunomodulating capacities (Li et al., 2015a; Han et al., 2019; Showalter et al., 2019). Besides, it has been recently described that even the type of culture as standard monolayer or as multilayers/globules (sometimes referred with the misleading names of 2D and 3D, respectively, since we live in a 3D world), may also modulate the properties of the exosomes produced by the MSC. Thus, human amnion-derived MSC grown in conditions to form aggregates or spheroids, produce more angiogenic and immunosuppressant factors than the ones grown in monolayers (Miceli et al., 2019). On the other hand, MSC pretreated with factors inducing the immune response, like interferon gamma (IFN-g) and TNF-a, generated EV with a higher immunosuppressing and/or immunomodulating capacity to direct the differentiation of M1 macrophages (pro-inflammatory) into the M2 (anti-inflammatory) phenotype (Domenis et al., 2018). Another study showed that EV derived from MSC at the latest stages of the osteoblastogenic induction had a higher capacity to induce osteoblastogenesis in undifferentiating MSC (Wang et al., 2018). Additionally, MSC manipulation, such as inducing the overexpression of miR-30b, enhanced the angiogenic capacity of their exosomes (Gong et al., 2017). In summary, the cell secretomes may be significantly modulated by the microenvironment, as well as the physiological and differentiating conditions of the producing MSC (Kusuma et al., 2017).

Therefore, the knowledge of all these modulating factors is of paramount relevance. That should allow to engineer MSC cultures for the desired objectives (Kusuma et al., 2017). Conversely, this scenario may complicate the large-scale production of EV for specific clinical applications. Thus, it may be difficult to generate homogeneous batches of EV. Both the cell origins and their physiological states may determine their contents, and thus their therapeutic properties (Rani et al., 2015). Therefore, new developments are needed to reach such objective for large-scale EV production and homogenization. That may require the use of genetic engineering of MSC. Fortunately, the knowledge of the human genome/transcriptomes to which we have contributed (Lario et al., 1997), together with new revolutionary technologies, like “clustered regularly-interspaced short-palindromic repeats” (CRISPR), should allow to reach such a goal (Amoasii et al., 2018; Lee et al., 2018b; Rees and Liu, 2018; Ryu et al., 2019). Yet, another putative handicap to overcome is that, although the EV are not cells, and therefore cannot generate tumors by themselves, their contents may induce neoplasia in cells of some patients. Curiously, the EV may have dual antagonic effects on tumoral cells. Thus, they can both activate or inhibit the proliferation and migration of tumoral cells (Zhou et al., 2018; Shojaei et al., 2019). It is therefore critical to determine the EV cargos, including their beneficial and putative unwanted side effects, before being routinely used in clinical therapies (Konala et al., 2016).

## Skin Wound Healing

The skin is the largest organ, accounting for 16% of the body weight, besides arguably being the most important as well, protecting the organisms from external aggressions, such as immaterial agents like sunlight [e.g., ultraviolet (UV) radiation], as well as physical ones, including both inorganic (e.g., abrasion) and organic or biological, like parasites (Kolarsick et al., 2011). The skin is made of three main layers: epidermis, dermis, and hypodermis. In turn, the epidermis has five sublayers (from outside to inside): corneum, lucidum, granulosum, spinosum, and basale. The epidermis is mainly made by keratinocytes (95%). They are proliferative in the stratum basale, differentiating and replacing the ones of top sublayers. Thus, they progressively lose they nuclei, take an ovoid shape, and eventually detach (Gantwerker and Hom, 2011). Such detachment helps to complete the physiological healing of skin wounds, besides being an effective way to get rid of ectoparasites, like some bacteria. The epidermis also has intussusceptions (invaginations), harboring hair follicles associated to sebaceous glands (pilosebaceous units) and sweat glands. The pilosebaceous unit is one of the locations containing epithelial stem cells, differentiating into basal keratinocytes. Therefore, they are essential for skin re-epithelialization. On the other hand, the dermis is located below the epidermis, receiving the main blood supply of the skin. Additionally, it contains most of the dermic appendages (integumentary system), like apocrine and eccrine glands, as well as hair follicles. In the dermis, a superficial or papillary dermis and a deeper reticular dermis are distinguished. In addition, there are interdigitations called dermal papillae, located between the dermis and epidermis (Gantwerker and Hom, 2011). The main cells of the reticular dermis are the fibroblasts. They produce the extracellular matrix (ECM), mainly made by collagen.

**FIGURE 4:**
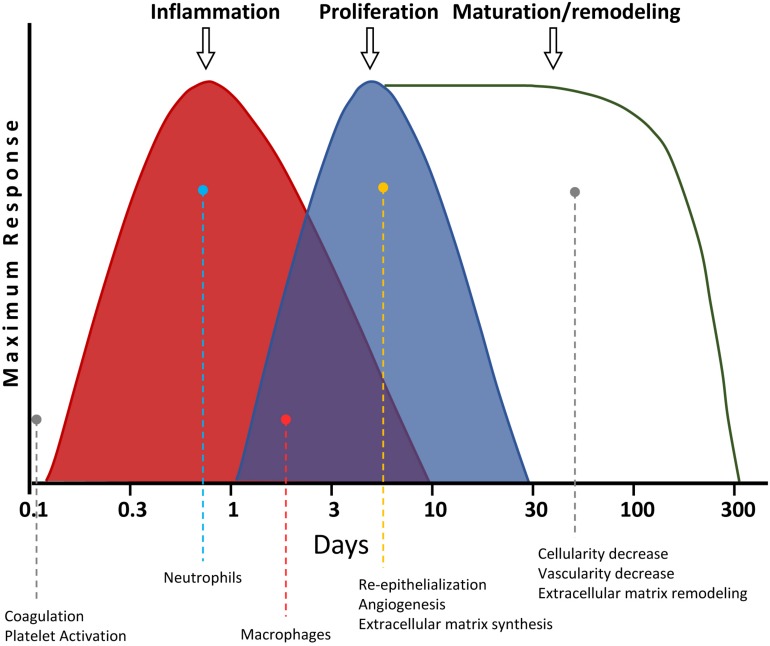
Timeline of skin wound healing. The different phases are shown. The most relevant processes for each stage, as well as their overlapping with others, are graphically and quantitatively depicted for each step.

There are four overlapping stages during skin wound healing: (i) hemostasis; (ii) inflammation; (iii) proliferation; and (iv) maturation/remodeling (Figure 4) (Gantwerker and Hom, 2011). The hemostasis is the first step, taking place a few seconds or minutes after the wound originates. The platelets produce a blood clot, preventing blood loss and entry of microorganisms. Besides, the platelets release different cytokines, hormones, and chemokines [e.g., PDGF, TGF-b, EGF, and fibroblast growth factor (FGF)], which are needed for the activation of the subsequent healing phases (Monaco and Lawrence, 2003), as described below. Then, the area of ulceration receives a stream of inflammatory cells during the second stage of wound healing. The first to arrive are the neutrophils, within the first 24 h after the wound is produced. These cells synthesize proteases and antimicrobial compounds, like ROS (Hart, 2002). After that, both the products generated by the neutrophils, as well as their initiation of apoptosis, attract macrophages and lymphocytes. They engulf and digest the remains of the matrix and cellular debris, as well as existing microorganisms, preventing infections. This way, the damaged zone gets cleaned. These events take place after about 48 h of wounding. The macrophages also release several cytokines at the end of this phase, which will activate regenerative processes in the next step (Robson et al., 2001; Hart, 2002).

New tissue is generated in such next proliferation phase. First, the re-epithelialization of the damaged skin takes place, due to the proliferation of the keratinocytes located at the edge of the wound. Additionally, an increase of VEGF induces angiogenesis from the blood vessels surrounding the wound. This way, the new generated tissue is vascularized. The fibroblasts also proliferate, producing a matrix of type III collagen, generating a granulation tissue. Some fibroblasts differentiate into myofibroblasts, with contractile function, effectively reducing the wound size, contributing to the wound closing. Thus, the wound scar becomes smaller than the original damaged area (Velnar et al., 2009). Finally, in the last maturation/remodeling phase, the ECM of type III collagen is replaced by other of type I collagen. Besides, many of the cells from the previous phase undergo apoptosis. This way, both the dermis cellularity and the blood vessel density are reduced. This last step takes longer than the others. After that, the tissue reaches its final appearance of healing (Gantwerker and Hom, 2011). The correct sequence and timing of these phases is fundamental for a proper wound healing. Thus, for instance, if the inflammatory phase is not carried out appropriately and takes more than 3 weeks, the ulcer may become chronic (Szpaderska and DiPietro, 2005). Among the main risks for that are aging, diabetes, and recalcitrant infections. Likewise, an excess of fibrosis may generate hypertrophic scarring, which in the most extreme scenarios may degenerate into keloids (Velnar et al., 2009; Eming et al., 2014).

Unfortunately, the number of skin ulcers requiring frequent caring and cures by practitioners and nurses are growing, mainly in the most economically developed countries. That is due to the increased population aging and higher prevalence of diabetes (Wild et al., 2004). The latter is reaching epidemic proportions worldwide, with 463 million people living with such disease, and therefore having higher risk of skin ulcers (International Diabetes Federation [IDF], 2019). This way, open wounds are a problem for 3% of the population older than 65 years of age in the USA. Besides, the USA Government estimates that the elderly population will be over 55 million by 2020, and thus, the number of chronic wounds is expected to grow (Sen, 2019). This represents a very important health expenditure for the national health systems of such countries. In particular, foot ulcers are associated to diabetes, having a prevalence between 15 and 25%; and the management cost of these ulcers is $9 milliards to $13 milliards in the USA (Raghav et al., 2018).

Taking into account the previous data, it is important to prevent the appearance of CSU. Likewise, to develop new and more effective treatments, accelerating their healing. A new approach with great potential is the deployment of cellular therapies, using MSC in the ulcerated zone, to favor its regeneration and healing (Kucharzewski et al., 2019). Indeed, numerous studies about this approach have produced encouraging results. They included both preclinic trials with animal models, such as mouse, rat, dog, or pig, as well as clinic ones with humans (Nuschke, 2014; Li et al., 2015b; Maranda et al., 2016). Both autologous and allogenous MSC from different origins have been used, including bone marrow, adipose tissue, umbilical cord, and the stromal vascular fraction, among others (Teng et al., 2014). Interestingly, several works have demonstrated that conditioned media from MSC cultures have a similar—or even higher—regenerative capacity than such cells, when applied to wounds. Therefore, the regenerative capacity of the MSC should be mainly due to their paracrine activity, as indicated above (Walter et al., 2010; Yew et al., 2011). For instance, it has been found that such conditioned media is more effective than full MSC to heal wounds of diabetic mice (de Mayo et al., 2017).

Besides, the secretome of these cells has been separated into the fraction containing soluble factors and metabolites, on one side, as well as the one made by EV, on the other side. Interestingly, the latter is the main activator of wound healing, as previously described. Thus, EV derived of bone marrow MSC increased migration and proliferation of dermal fibroblasts, and angiogenesis in human umbilical-vein endothelial cells (HUVEC). However, the exosome-depleted conditioned media had not such effects (Shabbir et al., 2015). Also, it has been shown that conditioned media from human adipose-derived stem cell (ADSC) cultures stimulated both the human dermal-fibroblast migration and closure of ischemic wounds in a rat model. That is due to the presence of long ncRNA (lncRNA) of metastasis-associated lung-adenocarcinoma transcript 1 (MALAT-1) in EV (Cooper et al., 2018).

## Application of MSC Extracellular Vesicles to Skin Wound Healing

The CSU are mainly characterized by an unfinished inflammation phase. As a consequence, the healing process does not end, stopping before an appropriate angiogenesis and tissue regeneration complete the physiological healing process. On the other hand, a defective wound healing, due to an increase in fibrosis, can cause hypertrophic scarring. Fortunately, the EV derived from the MSC have high immunomodulating, immunosuppressing, and angiogenic activities, as well as capacity to modulate the cellular proliferation and differentiation. Therefore, there are currently numerous researches evaluating the potential of these EV for the therapeutic treatment of the CSU and hypertrophic scarring. The works published at the time of writing were less than 50, and there is much to know about the practical clinical application of this therapeutic strategy. Yet, such published papers show that the EV derived from the MSC may positively modulate the different phases of the wound-healing process, as it has been proven by histological evaluations in animal models, including dogs (El-Tookhy et al., 2017).

### Inflammation

The EV are partially responsible for the immunomodulatory and immunosuppressant activities of the MSC. The proteomic analyses of EV from such cells have shown that they contain some cytokines, chemokines, and chemokine receptors, related to the immune system. They include interleukin 10 (IL-10), hepatocyte growth factor (HGF), leukemia inhibitory factor (LIF), chemokine (C-C motif) ligand 2 (CCL-2), vascular endothelial growth factor C (VEGF-C), and chemokine (C-C motif) ligand 20 (CCL-20), with immunosuppressive and regenerative activities. Besides, the cargo of the EV derived from the MSC may contain chemokine (C-X-C motif) ligand 2, 8, and 16 (CXCL-2, CXCL-8, and CXCL-16, respectively), defensin alpha 1 (DEFA-1), homologous to the E6-associated protein (E6-AP) carboxyl terminus (HECT), and regulator of chromosome condensation 1 (RCC-1)-like domain (RLD)-containing E3 ubiquitin protein ligase 5 (HERC-5) and interferon-induced transmembrane protein 2 (IFITM-2), which are chemoattractant proteins of immune cells. Therefore, the EV may activate a better protection against possible infections in damaged tissues (Mardpour et al., 2019). In fact, the treatment of cultures of peripheral-blood mononuclear cells with EV, derived from the MSC, reduced the production of the IL-1b and TNF-a inflammatory cytokines, while increasing TGF-b (Chen et al., 2016).

Interestingly, the effects of the EV derived from the MSC, on tissue inflammation, may be modulated by the environment in which the producing cells are growing. Thus, when they are kept in priming conditions, consisting of serum deprivation and cultured under hypoxic conditions (1% O_2_), the EV derived from them were enriched in some specific metabolites. They include adenosine, arginine, aspartic acid, cholesterol, glutamine, nicotinamide, uridine diphosphate (UDP) *N*-acetylglucosamine (UDP-GlcNAc), 5’-deoxy-5’-methylthioadenosine (MTA), palmitic acid, and isoleucine. Curiously, such molecules have been associated to anti-inflammatory activities, M2 macrophage polarization, and induction of regulatory T lymphocytes (Showalter et al., 2019). Besides, the EV obtained from the MSC, after being exposed to inflammatory cytokines (IFN-g and TNF-a) for 40–48 h, inhibited the proliferation of B lymphocytes and natural killer (NK) cells, after being intaken by them (Di Trapani et al., 2016). Other authors have stimulated MSC with such inflammatory cytokines, showing that the anti-inflammatory activities of their EV were partially due to their effects on the cyclooxygenase-2 (COX-2)/PGE2 pathway (Harting et al., 2018). Thus, such EV contained abundant COX-2, involved in the biosynthesis of PGE2. Actually, it has been demonstrated that, after tissue injury, the latter promoted an anti-inflammatory effect, favoring the inflammation resolution *in vivo* (Loynes et al., 2018). Such strategy of using EV from preconditioned MSC (grown in inflammatory environments) has been used in the treatment of chronic inflammation and wound healing, in experimental models *in vitro* and *in vivo*, respectively (Ti et al., 2015).

The first macrophages arriving to a wound exhibit the “classically activated” M1 phenotype. Then, a change to “alternatively activated” M2 phenotype takes place, due to signals from the microenvironment. The pro-inflammatory responses of the M1 macrophages are activated through Toll-like receptors (TLR), as well as the activation of nuclear factor kappa-light-chain enhancer of activated B cells (NF-kB). That leads to pathogen phagocytosis, oxidative burst, and intracellular killing. The activation of the M2 macrophages leads the one of the signal transducer and activator of transcription 3 (STAT-3), or other transcription factors. They inhibit inflammation and promote tissue remodeling (Liu et al., 2014). Unfortunately, the high glucose concentrations arising with diabetes prevent the correct polarization of M1 macrophages toward the M2. That generates a chronic inflammation, preventing a correct healing (Bannon et al., 2013). Some researchers have shown that the EV obtained from MSC, previously activated with lipopolysaccharides (LPS) (LPS pre-Exo), have a higher anti-inflammatory activity, inducing the macrophage polarization toward the M2 phenotype. Thus, the treatment of skin wounds in diabetic animals with LPS pre-Exo reduced the inflammation and accelerated the skin wound healing. These effects were in part due to a high miRNA let-7b expression in the LPS pre-Exo. Such miRNA inhibited the Toll-like receptor 4 (TLR-4), and therefore the inflammatory response. In fact, these authors detected a reduction of the NF-kB activation, besides an increase of the STAT-3 in cultures of a human acute monocytic leukemia cell line (THP-1), treated with LPS pre-Exo (Ti et al., 2015).

The concentration of TNF-a and IL-1b increased, whereas the IL-10 levels decreased, in a model of burn injury in rats, as well as in macrophage cultures exposed to LPS. The treatment with EV derived from human umbilical-cord MSC (hucMSC), but not with the ones derived from human skin fibroblast cells, reduced the inflammation in both cases. That happens through the suppression of the TLR-4 signaling pathway (Li et al., 2016). Interestingly, these authors demonstrated that such effects are due to the presence of miR-181c in the hucMSC-derived EV. Such miRNA is a repressor of the TLR-4 expression (Zhang et al., 2015c; Li et al., 2016).

### Angiogenesis

An appropriate regeneration requires the formation of new blood vessels, during the skin wound healing. That is a fundamental process for the delivery of oxygen, nutrients, and growth factors to the damaged tissues. A condition of hypoxia arises when the vascular tissue is damaged by a lesion. That activates the hypoxia-inducible factor-1 (HIF-1), which is a transcriptional activator, promoting the angiogenesis, by upregulating hundreds of target genes. Among them are the vascular endothelial growth factor A (VEGF-A) (Mole et al., 2009). It activates the endothelial cells of the surrounding blood vessels of the wound, generating new vessels. Other factors gear the growth of blood vessels, following the oxygen gradient (Okonkwo and Dipietro, 2017). Yet, unfortunately, the angiogenesis is reduced in the CSU of diabetics. That is partially due to the macrophages not polarizing toward M2. Since the latter are an important source of angiogenic factors, the chronic inflammation inhibits the angiogenesis in such patients. The EPC are mobilized from the bone marrow, to favor the angiogenesis after a tissular damage. But such cell population is decreased in diabetics (Okonkwo and Dipietro, 2017).

The angiogenic activity of the EV derived from the MSC has been investigated in numerous works, in relation to their application in the treatment of the CSU. Thus, HUVEC have been treated *in vitro* with EV obtained from MSC, derived from the adipose tissue. Interestingly, the results showed that the endothelial cells engulfed the EV, increasing the proliferation, migration, and angiogenesis events (Ren et al., 2019). These effects were accompanied by an upregulation of genes related to the proliferation (cyclin D1, cyclin D2, cyclin A1, and cyclin A2), angiogenesis [VEGF-A, platelet-derived growth factor subunit A (PDGF-A), EGF, and FGF 2 (FGF-2)], and migration [integrin beta 1 (ITGB-1) and CXCL-16]. The induction of the expression of these genes was associated to both, the activation of adrenocortical lipid-depletion with high leukemia incidence in mice due to AKV retrovirus (AKR) that develops thymomas (AKT), also known as protein kinase B (PKB), as well as the extracellular-signal-regulated kinase (ERK) signaling pathways in HUVEC. Besides, the treatment of skin wounds in mice with these EV produced an increase of the vascularization and proliferation of endothelial cells. The consequence was a healing acceleration, in relation to untreated wounds (Ren et al., 2019). Additionally, the EV derived from the MSC isolated from bone marrow can be engulfed by the HUVEC, enhancing their angiogenic capacity (Shabbir et al., 2015). Interestingly, it has been found that such EV carry the transcription factor STAT-3. It is involved in numerous cellular processes, like proliferation, migration, and angiogenesis. Thus, the mechanisms of action of the EV derived from the MSC on angiogenesis might be mediated trough the AKT/ERK/STAT-3 signaling pathways (Shabbir et al., 2015; Ren et al., 2019).

Another source of EV derived from MSC is provided by hiPSC-MSC. They can be generated from different adult cell types, after genetic manipulation. The EV derived from hiPSC-MSC have capacity to promote skin wound healing, collagen biosynthesis, and vascularization at wound sites in rats. Besides, they induce angiogenesis in HUVEC *in vitro* (Zhang et al., 2015b).

The proangiogenic capacity of the EV for regenerative medicine can be enhanced by preconditioning the producing cells. It is known that hypoxia is an angiogenic inducer (Han et al., 2019). Thus, MSC derived from bone marrow have been preconditioned with deferoxamine (DFO) for 48 h. That is a classical hypoxia-mimetic agent, which activates genes induced by hypoxia (Templeton and Liu, 2003). The EV derived from such cells (DFO-Exos) increased the proliferation, migration, and angiogenesis of HUVEC, in relation to unconditioned ones. In those EV, it has been identified miR-126 as an important angiogenic factor, being highly expressed in DFO-Exos. Such miRNA reduced the expression of the phosphatase and tensin homolog (*PTEN*) gene and activated the phosphoinositide 3-kinase (PI3K)/AKT signaling pathways. This way, the angiogenesis was enhanced. DFO-Exos were applied to skin wounds in a rat model induced to suffer diabetes with streptozotocin. Interestingly, they were more effective inducing angiogenesis, and accelerating wound healing, than the EV obtained from unconditioned MSC (Ding et al., 2019).

Yet another possibility to generate EV with high angiogenic capacity is to use genetic engineering (bioengineering), to design secretory cells producing factors that activate angiogenesis. On the other hand, the increase of oxidative stress is one of the factors preventing the CSU healing, mostly in diabetics (Wei et al., 2009). Additionally, the nuclear factor erythroid 2 (E2)-related factor 2 (NRF-2) is translocated from the cytoplasm into the nucleus in oxidative-stress conditions. This way, such transcription factor induced the expression of genes encoding antioxidant enzymes (Kensler et al., 2007). Therefore, the effects of EV from ADSC overexpressing the gene encoding NRF-2 have been studied, including an animal model of diabetic foot ulcers (Li et al., 2018). Thus, the administration of EV derived from ADSC to EPC maintained in high glucose, reduced the senescence, oxidative stress, and expression of inflammatory cytokines. Such effects, besides the angiogenesis, were enhanced when the EV come from ADSC overexpressing the gene encoding NRF-2. Besides, such EV significantly increased the formation of granulation tissue, angiogenesis, and the levels of growth-factor biosynthesis in a streptozotocin-induced diabetic rat model. Additionally, they reduced the levels of inflammation and oxidative stress-related proteins, as well as the ulcerated area in wound beds (Li et al., 2018).

Different studies have been carried out to better understand the mechanisms activating the angiogenesis during CSU healing, after being treated with EV. That includes the involvement of several miRNA and the activation of the AKT, ERK, and STAT-3 signaling pathways (Shabbir et al., 2015; Ding et al., 2019; Ren et al., 2019). Additionally, it has been demonstrated that the EV can also enhance the angiogenesis, through the portmanteau of “Wingless” and “Int-1” called “wingless-related integration site” (Wnt)/b-catenin (or canonical Wnt) pathway (Zhang et al., 2015a). Its activation is important for the endothelial function and healing (Goodwin et al., 2006; McBride et al., 2014). The EV derived from the hucMSC favored the proliferation, migration, and angiogenic capacity of the EA.hy926 HUVEC-derived line. Besides, they promoted the angiogenesis in a rat model of skin-deep second-degree burn (Zhang et al., 2015a). Such effect was mediated by the Wnt family-member 4 (Wnt-4) present in the EV, which activated the Wnt/b-catenin signaling in endothelial cells. Thus, EV derived from hucMSC, with silenced Wnt-4, did not activate the nuclear translocation of b-catenin, losing their angiogenic capacity (Zhang et al., 2015a).

The previous reports showed a positive action of the EV on angiogenesis, and therefore, on CSU healing. Yet, such cellular vesicles exhibited a rapid clearance rate *in vivo*, with a relatively short half-life. Therefore, it would be convenient to increase their stability, once applied *in vivo* (Wang et al., 2019a). Different approaches have been carried out to reach such a goal, and increase the EV effectiveness. For instance, a thermosensitive, injectable, self-healing, and adhesive polysaccharide-based fluorinated ethylene-propylene (FEP) hydrogel scaffold has been developed. The EV derived from the MSC isolated from adipose tissue were loaded into that gel. Such structure is named as FEP@exosomes (FEP@exo), releasing the cellular vesicles in a pH-dependent manner. Interestingly, the FEP@exo strategy increased the proliferation, migration, and angiogenesis of the HUVEC *in vitro*, accelerating wound healing in a diabetic mouse model. Indeed, the FEP@exo stimulated the angiogenesis, formation of granulation tissue, collagen deposition, and re-epithelization of the skin. The efficiency of this scaffold dressing approach is thought to be due to several reasons: (i) maintaining a wet environment; (ii) absorbing wound secretions; (iii) acting as antimicrobial agent; (iv) protecting against the UV light; and (v) favoring angiogenesis, through the liberation of EV (Wang et al., 2019a).

Other approaches using matrices in the wound bed, for the storage, protection, and liberation of EV with angiogenic capacity, have also been reported. They include the use of chitosan hydrogel. Indeed, it has been found that the administration of such linear polysaccharide in open wounds favored their healing. Besides, it is biocompatible and biodegradable, with antimicrobial activity, being a good carrier for sustained release of materials, such as EV (Ishihara et al., 2006). Researchers have used chitosan with exosomes derived from synovium-mesenchymal stem cells (SMSC), bioengineered to overexpress miR-126-3p (Tao et al., 2017). Such miRNA induced angiogenesis (Wang et al., 2008). The EV derived from the SMSC, expressing such miRNA (SMSC-126-Exos), exhibited interesting features. Thus, they induced the proliferation of human dermal-microvascular endothelial cell 1 (hDMEC-1) *in vitro*, as well as migration and capacity to generate tubular structures, through the activation of both the PI3K/AKT, as well as the mitogen-activated protein kinase (MAPK)/ERK pathways (Tao et al., 2017). Interestingly, the application of chitosan, loaded with SMSC-126-Exos, accelerated wound healing in rats with streptozotocin-induced diabetes. That was mainly due to enhanced angiogenesis. That demonstrates the exciting potential of EV enriched in proangiogenic factors for CSU healing, mainly using appropriate matrices (Tao et al., 2017).

### Fibroblasts

The dermal fibroblasts are one of the most important cell lines involved in the normal wound-healing. Their main functions in such a physiological process are ECM production, collagen biosynthesis, wound contraction, re-epithelialization, and tissue remodeling (Darby et al., 2014). If the fibroblasts do not act properly, an excessive production of ECM may occur, leading to scarring (Eming et al., 2014). Indeed, numerous studies have shown that the effects of the EV from MSC in wound healing are, at least in part, due to their capacity to modulate the fibroblast biology. Thus, cultures of fibroblasts from normal donors and chronic wound patients have been treated with EV from bone-marrow MSC. Interestingly, they increased the cellular proliferation and migration (Shabbir et al., 2015). Indeed, such fibroblasts exhibited an activation of the AKT, ERK 1/2 (ERK-1/2), and STAT-3 pathways. The latter transcription factor is active inside the MSC EV, being responsible for the induction of genes involved in cell-cycle progression in fibroblast cultures. They include the avian-myelocytomatosis virus oncogene cellular-homolog (c-MYC), cyclins A1 and D2, HGF, growth factors [insulin-like growth factor 1 (IGF-1), nerve growth factor (NGF), and stromal-derived growth factor-1 (SDF-1)], and interleukin 6 (IL-6) cytokine (Shabbir et al., 2015). The EV derived from MSC obtained from adipose tissue have also been tested on fibroblast cultures. They are engulfed by the cells, increasing their proliferation and migration. The treatments with these EV produced several beneficial effects for wound healing. Thus, they induced the expression of several genes, including c-MYC, matrix metallopeptidase 9 (MMP-9), EGF, FGF-2, TGF-b, vascular endothelial growth factor receptor (VEGFR), VEGF-A, and PDGF-A. Likewise, they increase the amounts of several proteins involved in such physiological activity. Among them were the endothelial growth-factor receptor 2 (VEGFR-2), cyclin D1, fibronectin, collagen I and III, and elastin. These effects were also mediated by an increase of the activation of the AKT and ERK signaling pathways (Ren et al., 2019).

Overall, the results obtained so far show that the fibroblasts, when are treated with EV from MSC, have a higher capacity to produce different factors. They favor the angiogenesis and biosynthesis of proteins for the ECM. Thus, the ones used in an *in vivo* model of skin ulcers significantly increased the collagen deposition (Ren et al., 2019). Other study, carried out with the MSC EV obtained from adipose tissue, showed that cultures of human skin fibroblast, treated with these EV, increased the cell proliferation and migration. Likewise, they upregulated the expression of genes encoding the cluster of differentiation 34 (CD-34), collagen type 1, elastin, and keratinocyte growth factor (KGF), which are related to skin regeneration. Interestingly, genomic studies of such EV identified hsa-miR-4484, hsa-miR-619-5p, and hsa-miR-6879-5p as the most expressed miRNA. They can regulate the expression of different genes, like nucleophosmin 1 (NPM-1), programmed cell death 4 (PDCD-4), chemokine (C-C motif) ligand 5 (CCL-5), and nucleoporin 62 (NUP-62). They were related to proliferation and aging, being therefore involved in tissue regeneration (Choi et al., 2018).

The mechanisms of action of the EV on the fibroblasts are carried out through several ways, including: (i) miRNA content; (ii) capacity to activate the AKT/ERK/STAT-3 pathways; and (iii) activation of the Wnt/b-catenin pathway. In relation to the latter, the effects of the EV derived from the hucMSC on fibroblasts and wound healing in a deep second-degree burn injury in a rat model were mediated by their high Wnt-4 content, which is an activator of the Wnt/b-catenin pathway (Zhang et al., 2015a). On the other hand, the hucMSC EV obtained from the acellular gelatinous Wharton’s jelly (structure envelope of the arteries and veins of the umbilical cord) (Meyer et al., 1983) have also been studied. Such EV enhance dermal fibroblast viability and migration *in vitro*, as well as skin wound healing in mice. It is interesting that these effects did not occur if the EV were lysed. Some authors have suggested that the EV must be intact when applied. The rationale is that intact EV allow the interaction between membranes and/or ensures that their cargos are delivered with enough concentration. This study also described that the effects of the EV were due, at least in part, to their high content of alpha-2-macroglobulin. Nevertheless, they did not identify the mechanisms by which this protein may act in wound healing (Bakhtyar et al., 2018).

Interestingly, EV derived from fetal dermal-mesenchymal stem cells (FDMSC) have been isolated from fetal skin. A potential advantage of such cells in regenerative medicine is their scarless wound-healing capability (Leavitt et al., 2016). Indeed, the EV derived from the FDMSC accelerated wound closure in a mouse full-thickness skin wound model. Besides, they increased the proliferation and migration of dermal fibroblasts *in vitro*. In addition, they induced the expression of genes encoding ECM proteins, such as collagen type I and III, fibronectin 1, elastin, and alpha-smooth muscle actin (a-SMA) (Wang et al., 2019b). These authors have shown that the effects of such EV on fibroblasts were mediated by activation of the Notch pathway. That was a consequence of the EV carrying the Notch ligand, known as jagged 1 (JAG-1) (Wang et al., 2019b). That is in agreement with the important roles assigned to the Notch-signaling pathway in wound healing (Chigurupati et al., 2007; Shi et al., 2015).

It is important to take into account that the fibroblasts may play a critical role in scar formation, during wound healing. Indeed, an excessive production of ECM may favor such unwanted side effect. This can be prevented by applying EV (Zhao et al., 2017). Thus, the intravenous administration of EV, derived from human MSC from adipose tissue, in a mouse model, increased the expressions of collagen type I and III after 5 days of treatment, being reduced after 14 and 21 days of such administration. These results suggest that the EV may promote the early stages of wound healing, while inhibiting collagen biosynthesis—effectively reducing scar formation—at the later stages (Hu et al., 2016). More recent works of the same research team have shown that the administration of such EV promoted the ECM remodeling, as well as the scarless healing of cutaneous wounds. Yet, such effects were not found when using EV-free conditioned medium (Wang et al., 2017). These authors have demonstrated that the *in vivo* treatment with EV produced interesting results: (i) increase ratios of collagen type III:I, TGF-b 3:1 (TGF-b3:TGF-b1) and stromelysin-1 (also known as matrix metalloproteinase 3):tissue inhibitors of metalloproteinase/metallopeptidase inhibitor 1 (MMP-3:TIMP-1); and (ii) reduction of the differentiation of fibroblasts into myofibroblasts. Interestingly, such processes favored a proper remodeling of the ECM, being similar to the ones found in fetal wound healing, in which scars were not produced. Besides, *in vitro* studies have shown similar results when cultures of dermal fibroblasts were treated with EV. These effects were mediated by the MAPK/ER pathway. These results suggest that the activation of this signaling pathway by EV was, at least in part, responsible for scar reduction (Wang et al., 2017). Additionally, EV derived from the adipose tissue accelerated wound healing and reduced scar formation in full-thickness skin wounds, in a mouse model. Such authors also observed that the human dermal fibroblasts treated with such EV activated the biosynthesis of collagen type I and III, being the latter 50% higher than the former. On the other hand, they reported that the EV inhibited the biosynthesis of the a-SMA protein (Zhang et al., 2018). Those results are in agreement with the ones reported by other authors (Wang et al., 2017; Dalirfardouei et al., 2019). Therefore, the EV derived from the MSC of adipose tissue reduced scar formation, due to their effects on fibroblasts. Indeed, it has been found that such effects were mediated by the activation of the PI3K/AKT signaling pathway (Zhang et al., 2018).

On the other hand, the CSU associated to aging have been related, at least in part, to the beginning of senescence of dermal fibroblasts. That was due to both the chronic inflammatory status of this kind of ulcers, as well as a consequence of the aging effects (Harding et al., 2005; Wall et al., 2008). Fortunately, two interesting results have been recently reported. Thus, it has been found that: (i) the EV derived from embryonic stem cells of mice were enriched in mmu-miR-291a-3p; and (ii) such miRNA inhibited the senescence induced in human fibroblast cultures by replicative senescence, adriamycin-induced cellular senescence, or ionizing radiation-induced cellular senescence (Bae et al., 2019). Such latter effect was carried out through the repression of the gene encoding the transforming growth factor-beta receptor 2 (TGFBR-2). Its downregulation reduced the senescence in fibroblasts (Tsai et al., 2018; Bae et al., 2019). Besides, the treatment with mmu-miR-291a-3p accelerated *in vivo* skin wound healing in 12-month-old mice, but not in 8-week-old ones. Interestingly, the hsa-miR-372-3p and hsa-miR-371a-3p human miRNA (corresponding to mmu-miR-291a-3p in mouse) also had anti-senescence effects in fibroblasts. That opens the door to the use of EV enriched in such miRNA, for the treatment of skin ulcers associated to aging and diseases like diabetes (Bae et al., 2019).

### Keratinocytes

The keratinocytes intervene in the re-epithelialization of skin. That is considered as one of the major processes in wound healing (Escámez et al., 2004). Such cells begin to migrate from the wound edge into the wound gap, at very early stages of healing. His migration is independent of the granulation-tissue formation and can be considered the first step in normal healing. That provides the basis for the subsequent stages. The keratinocyte proliferation and differentiation in the wound-healing process take place in both parallel and sequential manners (Braiman-Wiksman et al., 2007). Thus, these cells are hyperproliferative and mitotically active at the edge of chronic wounds. But they fail to migrate and create a hyperkeratotic tissue at the edge of the wound (Stojadinovic et al., 2012; Martin and Nunan, 2015).

The MSC-derived EV increased the skin re-epithelialization in full-thickness cutaneous wounds in animal models (Robson et al., 2001; Zhang et al., 2015a, 2018; Li et al., 2017; Tao et al., 2017; Ren et al., 2019; Wang et al., 2019b). However, there are few works analyzing the specific effects of such EV in keratinocytes. It has been found that the so-called “human, adult, low calcium, high temperature” (HaCaT) skin keratinocyte cells, internalized EV derived from adipose-tissue MSC. In addition, those EV promoted the proliferation and migration of HaCaT, with an upregulation of proliferative marker genes, such as cyclins A1, A2, D1, and D2, as well as fibronectin. Interestingly, the latter were involved in cell adhesion (Ren et al., 2019). They also increased the levels of PDGF-A, VEGF-A, FGF-2, EGF, and VEGFR-2. Such results suggest that the EV treatments may contribute to angiogenesis in wounds, by means of the paracrine pathways, as previously described. Thus, the effects of EV on HaCaT seem to be due to the activation of AKT and ERK signaling pathways (Ren et al., 2019). Additionally, the application of EV from iPSC-derived MSC (iMSC) increased the proliferation and migration of HaCaT cultures, as well as ERK-1/2 activation (Kim et al., 2018).

The AKT pathway has also been involved in the positive effects of the hucMSC-derived EV in keratinocytes. Thus, wounds of a deep second-degree burn-injury model in rat have been treated with such EV. A significant upregulation of the gene encoding the cytokeratin 19 (CK-19) was found after 1 week of treatment. That is an epithelial biochemical marker biosynthesized by basal cells, on the external root sheath of hair follicles (Michel et al., 1996). Interestingly, a complete epidermal structure, in the CK-19-positive area of the wounds, was formed after 2 weeks of application. This shows that the treatment with hucMSC-derived EV promoted the skin re-epithelization. On the other hand, the apoptosis of HaCaT cultures, subjected to heat stress (43°C for 40 min), decreased when they were treated with hucMSC-derived EV. In addition, both cell proliferation and migration increased. These effects were mediated by both AKT and Wnt/b-catenin pathway activation, but in an independent manner. Such authors concluded that the activation of the AKT pathway can be induced by cytokines delivered by EV, such as the platelet-derived growth factor B-homodimer (PDGF-BB), granulocyte-colony stimulating factor (G-CSF or GCSF), VEGF, monocyte-chemoattractant protein 1 (MCP-1), as well as IL-6 and -8. On the other hand, b-catenin would be activated by the presence of Wnt-4 in the EV. The activation of both pathways would increase wound healing (Zhang et al., 2015a).

## Concluding Remarks and Future Prospects

The EV secreted by the cells play a significant role in both the intercellular communications, as well as the interactions with the cellular microenvironments. Such discoveries are fascinating and with great potential, allowing to exploit them to develop new and efficient therapeutic tools, for many pathologies. As described in this critical review, the properties of the EV depend on their cargos, which are confined and protected within a lipidic bilayer. That allows the EV to reach distant locations from the production site.

**FIGURE 5:**
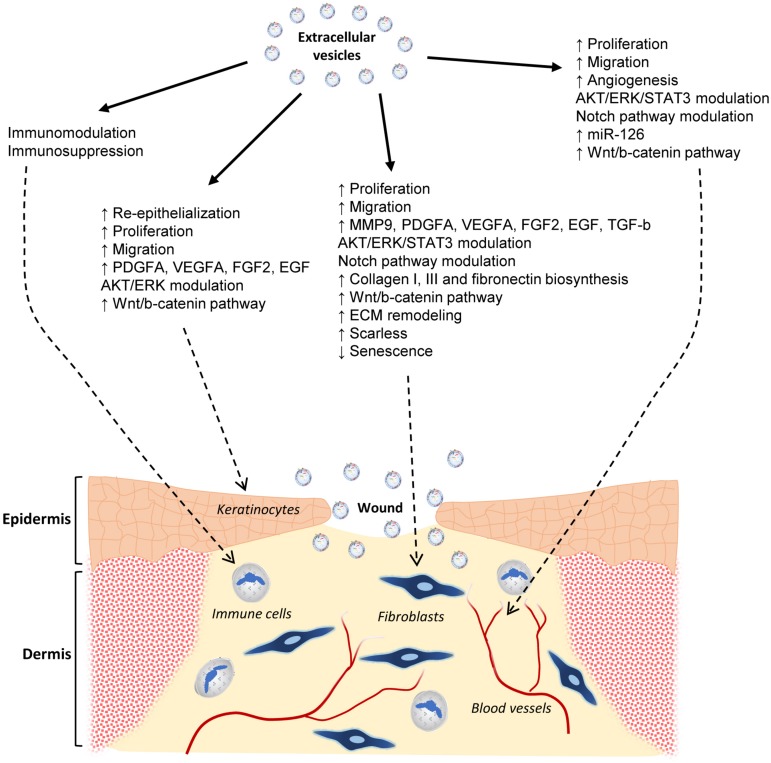
Effects of MSC extracellular vesicles on wound healing. Graphical abstract of the current state-of-the-art knowledge of the EV actions on skin wound healing. The effects on different cell types involved in such a physiological process are shown. They include immune and endothelial cells, fibroblasts, and keratinocytes.

The EV obtained from MSC are characterized by having immunomodulatory and regenerative properties, similar to the ones of the producing cells. They can be used in cell-free therapies in regenerative medicine, and more specifically, in CSU treatments. The currently available publications show that the EV can modulate different stages of wound healing. They include inflammation, angiogenesis, proliferation, and ECM remodeling. This way, they can effectively accelerate and overall improve the wound healing process (Figure 5). Nevertheless, although the current results are certainly encouraging, further research is still needed to optimize the EV applications, as routine clinical tools in CSU treatments. That includes their production, isolation, characterization, and ways of administration.

It is critical to determine the optimal source of MSC, if they should be bioengineered to produce the desired cargos in high amounts, as well as the putative requirement for cell immortalization. The latter is fundamental, since the cell senescence due to culture passages and time may alter the EV cargos, and, therefore, their properties. Also important is to determine the optimal culture conditions, depending on the experimental, clinical or therapeutic objectives to be reached, like wound healing. As described in this critical review, the cell preconditioning, for instance, in inflammatory or hypoxic environments, may significantly enhance and optimize the EV immunosuppressing or angiogenic capacities. Additionally, the methodology to isolate and purify the EV must be highly controlled and optimized. That is required to increase its efficiency, maintain the structure and functionality of such EV. Some events should be completely avoided. For instance, the putative contamination with other undesired particles, including cell components and culture media. The current methods of obtaining EV for skin wound healing are numerous. That makes difficult to properly compare them in many cases.

On the other hand, the characterization of the EV contents is also fundamental, to better understand their molecular mechanisms of action. Likewise, to predict putative unwanted side effects that their application may trigger. Numerous mechanisms of exosome action have been proposed in the available bibliography, depending on the specific study. For instance, they may involve the AKT, ERK, Notch, STAT-3, and Wnt/b-catenin signaling pathways, among others. Besides, different miRNA and other molecules may be involved. Interestingly, the current data show that similar skin wound healing results may be obtained from different pathways. Therebefore, many of the described mechanisms may be bound together, working in a coordinated, complementary, and interrelated way. That is not surprising, taking into account the exquisite integration of cellular functions allowing homeostasis and life itself. Nevertheless, a better understanding of their molecular bases is required. That is of paramount importance to optimize the EV cargo, which can be specifically designed by genetic engineering of the sourcing cells. It should also be taken into account that their contents may also be modulated by the tissue culture conditions. Besides, a better understanding of the molecules that can induce the acceleration of CSU healing should allow the future specific design of tailor-made particles. They could be similar—and even better—than the natural biological EV. Such a goal could be reached exploiting the tremendous power and flexibility of biotechnology and nanotechnology. These artificial constructs may become new drug carriers, with specific contents and targets. Actually, there are already exciting and promising results in the development of molecular origami based on DNA, RNA, and proteins, to carry drugs to specific cellular targets (Pugh et al., 2018). Finally, it is important to optimize the application of EV for the healing of skin wounds. Usually, they are subcutaneously applied in the wound edges. Sometimes, they are intravenously administered. That takes advantage of the property of these vesicles for targeting and concentrate in damaged tissues/cells. In other instances, the EV are liberated from a hydrogel placed over the wound. Therefore, it would be convenient to analyze the advantages and disadvantages, critically comparing the different application methodologies for each specific clinical goal. In principle, the use of EV embedded, in hydrogels or dressings with healing properties, has a great future potential for skin wound healing.

In conclusion, the currently available data on EV shed new light on this exciting topic and draw optimist prospects for their efficient exploitation in regenerative medicine, including the treatment of CSU. Nevertheless, further research is needed for a better understanding the molecular bases underlying these processes; and specifically on how the EV derived from MSC work on wound healing. Likewise, optimized methods for large-scale production of EV for human clinical applications must be developed. All such insights should have very positive impacts on regenerative medicine, as well as on the quality of life of the patients suffering different diseases. That includes the CSU in diabetics, among others. Indeed, some companies are focusing their investments efforts on the development of exosomes as therapeutics (Zipkin, 2019). And last but not least, that should also significantly reduce the overhead and cost of the private and public health and welfare systems worldwide.

## Author Contributions

AC-D designed the study. AC-D, GD, and JQ-G drafted, conducted reviews, literature analyses, edited, and approved the manuscript.

## Conflict of Interest

The authors declare that the research was conducted in the absence of any commercial or financial relationships that could be construed as a potential conflict of interest.

## Footnotes

1 https://www.ncbi.nlm.nih.gov/pubmed

2 https://systembio.com/products/exosome-research/exosome-isolation

3 http://www.clinicaltrials.gov
